# Modeling and measurement of lead tip heating in implanted wires with loops

**DOI:** 10.1088/1361-6560/adcc73

**Published:** 2025-04-22

**Authors:** Lydia J Bardwell Speltz, Seung-Kyun Lee, Yunhong Shu, Matt A Bernstein

**Affiliations:** 1 Department of Radiology, Mayo Clinic, Rochester, MN, United States of America; 2 GE HealthCare Technology & Innovation Center, Niskayuna, NY, United States of America

**Keywords:** implanted wires, RF heating, MR safety, loop, transfer function, adapted transmission line model

## Abstract

*Objective*. In MRI, conductive lead tip heating caused by radiofrequency (RF) power deposition is an important safety issue for patients with implanted devices. In this work, we investigate lead tip heating in different wire configurations that contain loop(s) through theoretical models and experimental measurements. *Approach.* We have previously proposed analytical transfer function models to predict relative heating of implanted, straight metallic leads. Here we extend the models’ application to leads containing loops, that are widely used in the clinic. Maximum temperature rise caused by RF heating was measured at 1.5 T on twenty (20) insulated, capped wires with various loop and straight segment configurations. The experimental results were compared with predictions from the previously reported simple exponential and adapted transmission line models, as well as with a long-wavelength approximation. *Main results.* Both models effectively predicted the trends in lead tip temperature rise for all the wire configurations, with the adapted transmission line model showing superior accuracy. In a typical MRI configuration where the RF electric field is predominantly in the superior/inferior (S/I) direction, wires oriented in the same direction showed decreased heating as the number of loops increased. However, when wires were oriented right/left (R/L) where the corresponding component of the electric field is negligible, additional loops increased the overall heating. *Significance.* The simple exponential and the adapted transmission line models previously developed for, and tested on, straight wires require no additional terms or further modification to account for RF heating in a variety of loop configurations. These results extend the models’ usefulness to manage implanted device lead tip heating and provide theoretical insight regarding the role of loops and electrical lengths in managing RF safety of implanted devices.

## 1. Introduction

Radiofrequency (RF) power deposition at the lead tips can cause substantial heating, posing a major safety concern for patients with implanted devices undergoing MRI examinations (Yeung *et al* 2002a, Nyenhuis *et al* 2005, Park *et al* 2007). Many of these devices are specifically designed and tested for scanning at a magnetic field strength of 1.5 T, which is commonly used clinically for patients who have implanted devices. Most active implants, including cardiac implantable electronic devices, deep brain stimulators, spinal cord stimulators, etc, contain an implantable pulse generator (IPG) and one or more leads. When leads are surgically implanted, their trajectories often include single or multiple loops of various sizes. Understanding how loops influence lead tip heating remains an ongoing area of research (Baker *et al* 2005, Mattei *et al* 2006, Golestanirad *et al* 2017, 2019, Bhusal *et al* 2021, Jiang *et al* 2023, Vu *et al* 2023). A model that can be applied to predict lead tip heating with various lead configurations could potentially inform lead placement strategies and improve safety protocols, i.e. allow greater access to MRI for patients with implants without increasing the risk of adverse effects.

Several studies investigating the impact of loops on lead tip heating have primarily focused on deep brain stimulation leads. Main findings from these studies are: (1) increasing the number of loops tends to *reduce* heating (Baker *et al* 2005, Golestanirad *et al* 2017, Bhusal *et al* 2021, Vu *et al* 2023), (2) positioning loops near the burr hole decreases heating (Baker *et al* 2005, Golestanirad *et al* 2019, Vu *et al* 2023) and (3) contralateral leads experience more heating than ipsilateral leads (Golestanirad *et al* 2019). A limited number of studies on pacemaker or epicardial leads have similarly found that: (1) increasing the number of loops also reduces heating (Mattei *et al* 2006), and (2) anterior-facing loops generate more heating than inferior-facing loops (Jiang *et al* 2023). The latter finding is not surprising because the *z*-component of the B1-field is designed to be relatively small. A recent study investigated heating in peripheral nerve and spinal cord stimulators by measuring the transfer function and observed that in phantom experiments and human body model simulations loops can either increase or decrease RF-induced heating, depending on the location of the loop (Akter *et al* 2024).

In a recent paper (Bardwell Speltz *et al* 2024), our group introduced a simple exponential model and an adapted transmission line model for the transfer function to predict lead tip voltage, and experimentally tested the models by measuring lead tip temperature rise in a series of straight, insulated wires. In this paper, we measure lead tip heating for a series of insulated wires that include both a straight section and loops of various size, number, and placement. We then applied the two models from (Bardwell Speltz *et al* 2024) to test whether they still hold in their original form, or whether they require modification to account for the presence of loops. We also considered a long-wavelength approximation to the two models (i.e. setting the transfer function equal to 1) that allows derivation of analytical results, which may provide some further insight. The objective of this study is to validate and potentially extend the existing analytical models to better predict lead tip heating in more complex lead configurations, specifically containing loops.

## 2. Theory

### 2.1. Transfer function models

Lead tip voltage can be calculated using the complex line integral of the incident electric field multiplied by a suitable transfer function *h* (Park *et al* 2007):

\begin{align*}V = \mathop \smallint \limits_0^d h\left( l \right){ } \boldsymbol{E}\left( l \right) \cdot {\text{d}}\boldsymbol{l}\end{align*}

where *l* is the distance from the lead tip, $d{\text{ }}$ is the total length of the lead, **E** is the electric field (which can be complex), and bold font indicates a vector. The line integral is frequently expressed using the tangential component of the electric field, ${E_t}$, which can also be complex (Park *et al* 2007, Feng *et al* 2015). For a short segment of wire whose path is given by a real vector $\Delta {\boldsymbol{l}}$, *E_t_* is defined as

\begin{align*}{E_t}\left( l \right) = \frac{{\boldsymbol{E}\left( l \right) \cdot \Delta {\boldsymbol{l}}}}{{\left\| {\Delta {\boldsymbol{l}}} \right\|}}.\end{align*}

The equation below shows how the predicted lead tip temperature rise ${{\Delta }}T$ can be calculated (Park *et al* 2007, Wang *et al* 2022) from the absolute square of the voltage, up to an unknown scaling factor:

\begin{align*}{\text{ }}\Delta T \propto {\left| V \right|^2}.\end{align*}

The transfer function contains a complex wavenumber (referred to here as King wavenumber) that takes into account the wavenumber of tissue (${k_t}$) and the wavenumber of the insulator (${k_i}$) that surrounds the lead conductor (King *et al* 1974). The equation for ${k_t}$ is shown below where conductivity ($\sigma $ in S m^−1^), permeability ($\mu $ in H m^−1^), and permittivity ($\varepsilon $ in F m^−1^) characterize biological tissue.

\begin{align*}{\text{Re}}\left({k_t}\right) = \omega \sqrt {\frac{{\varepsilon \mu }}{2}} \,{\left[ {\sqrt {1 + {{\left( {\frac{\sigma }{{\varepsilon \omega }}} \right)}^2}} + 1} \right]^{\frac{1}{2}}},\,{\text{Im}}\,\left({k_t}\right) = \omega \sqrt {\frac{{\varepsilon \mu }}{2}} \,{\left[ {\sqrt {1 + {{\left( {\frac{\sigma }{{\varepsilon \omega }}} \right)}^2}} - 1} \right]^{\frac{1}{2}}}.\end{align*}

In the equation above, $\omega {\text{ }}$ is the Larmor angular frequency (in units of rad s^−1^) and $\mu {\text{ }}$ can be approximated by the permeability of free space, 4$\pi \times {10^{ - 7}}$ H m^−1^. Because its conductivity $\sigma = 0,{\text{ }}$ the wavenumber in the insulator ${k_i}$ reduces to ${k_i} = $ $\omega \sqrt {{\varepsilon _i}\mu } $, a real number that depends on the permittivity ${\varepsilon _i}$ of the insulator. Using the wavenumbers of the tissue and the insulator, the King wavenumber can be determined (King *et al* 1974, Nitz *et al* 2001, Yeung *et al* 2002a, 2002b, Liu *et al* 2018, Bardwell Speltz *et al* 2024):

\begin{align*}{ }k = {k_{\text{R}}} + i{k_{\text{I}}} = {k_i}{\left( {1 + \frac{{F\left( {{k_t}b} \right)}}{{{\text{ln}}\left( {b/a} \right)}}} \right)^{1/2}},{ }F\left( z \right) = \frac{{H_0^{\left( 1 \right)}\left( z \right)}}{{z{ }H_1^{\left( 1 \right)}\left( z \right)}}{ }\end{align*}

where ${k_{\text{R}}}$ and ${k_{\text{I}}}$ are the real and imaginary components. Here *a* and *b* are the inner and outer radii of the insulator, respectively. $H_0^{\left( 1 \right)}\left( z \right)$ and $H_1^{\left( 1 \right)}\left( z \right)$ are Hankel functions of the first kind of order 0 and 1, respectively (Milton and Stegun 1972). To ensure ${k_{\text{I}}} \le 0$, which means the imaginary part of the wavenumber produces losses, we make a sign choice for the square root in equation (5). The King wavenumber is used in the two forms of the transfer function considered in Bardwell Speltz *et al* (2024). The simple exponential model is given by,

\begin{align*}h\left( l \right) = { }{{\text{e}}^{ - {\text{i}}kl}}.\end{align*}

This model is used to describe the simplest transfer function that still accounts for resonant length and loss effects.

The adapted transmission line model is given by,

\begin{align*}\begin{array}{*{20}{c}} {h\left( l \right) = \frac{{{{\text{e}}^{ - {\text{i}}kl}}\left( {1 - \Gamma {{\text{e}}^{ - 2{\text{i}}k\left( {d - l} \right)}}} \right)}}{{\left( {1 - \Gamma {{\text{e}}^{ - 2{\text{i}}kd}}} \right)}}{ }} \end{array}.\end{align*}

A transmission line model was described by Liu *et al* (2018) using the principle of reciprocity. Equation (7) takes into account the voltage wave that is reflected from the IPG end of the lead (Liu *et al* 2018), with a reflection coefficient $\Gamma $. Equation (7) reduces to equation (6) when $\Gamma = 0$, and is normalized to $h\left( 0 \right) = 1$.

**Figure 1.**
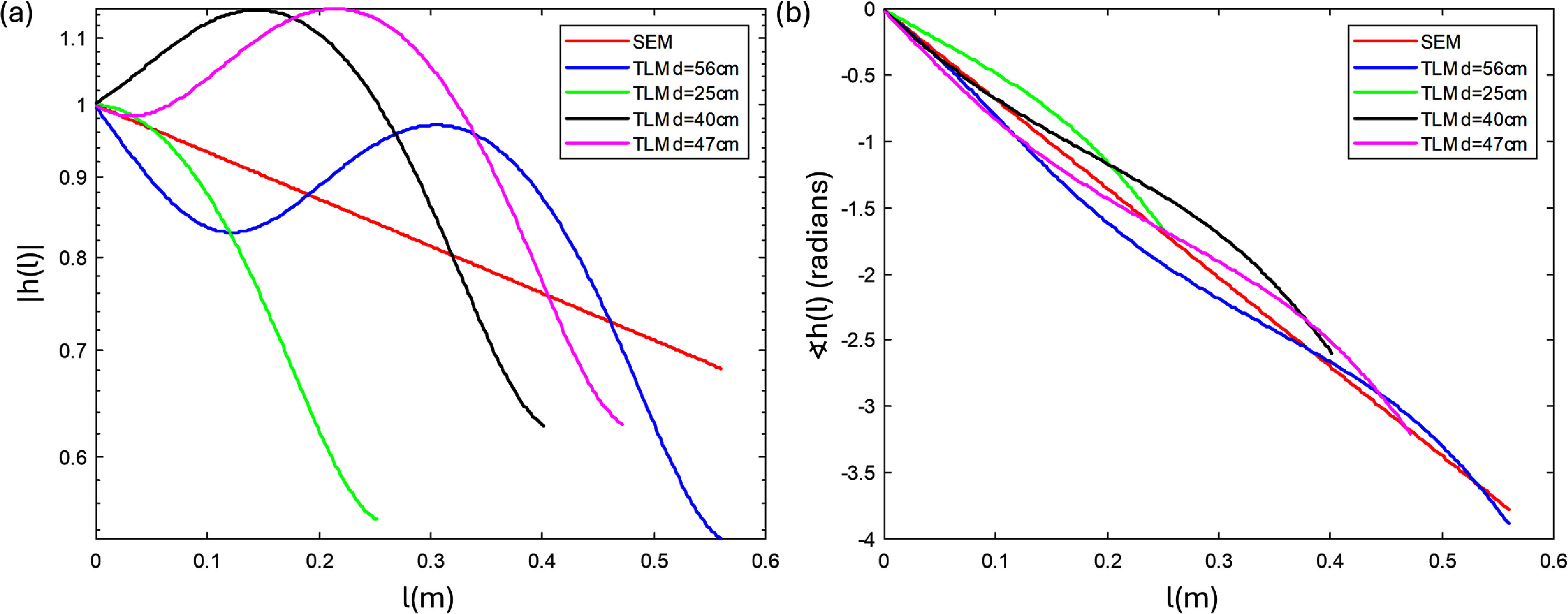
The (a) magnitude and (b) phase of the simple exponential (SEM) and adapted transmission line (TLM) transfer functions $h\left(l\right)$ plotted versus the length variable $l$. Log scale is used on the vertical axis in (a). The adapted transmission line transfer function depends on *d* (total wire length) so the four TLM lines with different *d* have qualitatively different functional dependencies on $l$. Nevertheless, they all exhibit overall decaying trend with $l$ governed by Im(*k*) < 0.

The insulated wires used in this work had inner and outer radii of the insulator $a{\text{ }}$ = 0.390 mm and $b{\text{ }}$ = 0.625 mm, resulting in a calculated King wavenumber of (6.7454–0.6840) rad m^−1^ at 1.5T. To calculate the temperature rise $\Delta T \propto {\left| V \right|^2}$ from equation (1) for a set of wires, the simple exponential model requires only one free parameter per set of data for global scaling, while the adapted transmission line model requires a second free parameter to fit the reflection coefficient (unless these parameters are obtained by other means, such as modeling). Both models predict the resonant length without the use of any free parameters. Figure 1 shows plots of the magnitude (with a log scale on the *y*-axis) and phase of the transfer function for the simple exponential model and the adapted transmission line model for various values of length *d*, and the specific value of the wavenumber *k* we considered. The reflection coefficient was set to ${{\Gamma }}$ = 0.2403 based on experimental data fitting (see Results).

### 2.2. Effect of loops

One approach to understand the effect of loops in equation (1) is to apply the well-known Stokes’ theorem (Arfken 1985), which states the line integral of a function over a closed loop defining a bounded surface is equal to the surface integral of the curl of the function. This theorem can be applied in conjunction with one of Maxwell’s equations (Jackson 1999):

\begin{align*}\nabla \times {\boldsymbol{E}} = - \frac{{{\text{d}}{\boldsymbol{B}}}}{{{\text{d}}t}}\end{align*}

to yield Faraday’s law of induction. If the magnetic field has sinusoidal time dependence represented by a complex exponential, then the right-hand side of equation (8) becomes $ - i\omega {\boldsymbol{B}}$, further simplifying the calculation.

Equation (8) can be used to estimate the contribution of a loop in the line integral equation (1) provided $h\left( l \right)$ can be assumed constant *over the length of the loop* so that it can be removed from the integral. This is satisfied if the King wavelength is much larger than the loop’s circumference, which is roughly true in our case where the former is $2\pi /\left| k \right|$ = 93.1 cm and the largest loop diameter was 7 cm.

In this case, the part of the line integral in equation (1) over a loop with area *A* can be replaced, up to a constant factor, by equation (8) multiplied by *A*, yielding a voltage magnitude *V*_loop_ = $A{\text{ }}\omega {\text{ }}b1$, where *b*1 is the average RF magnetic field amplitude over the loop. This can be compared with the line integral of the same section of wire if it were straight rather than forming a loop. While exact calculation depends on the geometry (see appendix), dimensional analysis suggests that such quantity takes the form ${L_1}{\text{ }}{L_2}{\text{ }}\omega {\text{ }}b1$, where *L*_1_ and *L*_2_ are relevant length scales of the problem. Given that the line integral is taken along the length of the wire and the RF *E* field generally increases with distance from the isocenter, *L*_1_, *L*_2_ can reasonably be replaced by *d*_section_ (length of the wire section) and *x* (representative distance from the isocenter). This gives the induced voltage over the straight section of the wire as ${V_{{\text{straight}}}} \approx { }x{ }{d_{{\text{section}}}}{ }\omega { }b1$. Since $x{ }{d_{{\text{section}}}} \gg A$ in most practical loop placements, it is expected that a loop reduces the contribution to the lead tip voltage compared to a straight wire section.

If the same section of wire is configured into multiple (*N* > 2) loops, equation (8) is multiplied by the number of loops, *N*, but the area of each loop decreases by a factor of *N*^2^. The net effect is therefore 1/*N* smaller voltage contribution, predicting a beneficial effect of multiple loops.

In the appendix, we derive exact expressions for ${V_{{\text{loop}}}}$ and ${V_{{\text{straight}}}}$ under simplifying assumptions of $h = 1$ and uniform RF magnetic field. While simplified, the analytical model confirms our general estimation given above and predicts results consistent with empirical findings on the benefit of loops in lead tip heating (Baker *et al* 2005, Golestanirad *et al* 2017, Bhusal *et al* 2021, Vu *et al* 2023).

We emphasize that the equations in the appendix were not used to calculate voltage in the following sections. Instead, we numerically evaluated equation (1) for each lead configuration as described in Methods. This is because the simplified analysis of the appendix fails to explain some other previously reported experimental findings (Baker *et al* 2005, Golestanirad *et al* 2019, Vu *et al* 2023), e.g. that different placement of the loop along the lead can affect the observed temperature rise. Specifically, it was reported consistently that loops placed near the tip (or the burr hole) reduced heating better than loops farther away from the tip. Consequently, we return to the more realistic transfer functions of equations (6) and (7) and the simulated electric field described in section 3.3, to examine how well they model those effects.

Findings related to the positioning of the loop can be understood in terms of the concept of ‘electrical length,’ which is captured in the transfer function $h\left( l \right)$. In this interpretation, a wire with loops positioned closer to the lead tip are expected to heat up less than a wire with equivalent loops farther away from the tip. This is because the former loops add path length, which attenuates the transfer function (see figure 1) over a greater proportion of the non-looped path of the line integral.

The goal of the experimental part of this paper is to confirm that equation (1), in conjunction with a suitable transfer function, can provide quantitative predictions related to three of the findings reported in the literature on the role of loops in lead tip heating: (i) loops reduce heating in general, (ii) more turns, while keeping the total lead length fixed, also reduces heating, and (iii) loops closer to the lead tip tend to result in reduced heating. Through a set of controlled wire heating experiments, we test how well such model predictions agree with measured data, providing insight into the physical mechanism of how loops alter lead tip heating.

## 3. Methods

### 3.1. American society for testing and materials (ASTM) phantom

**Figure 2.**
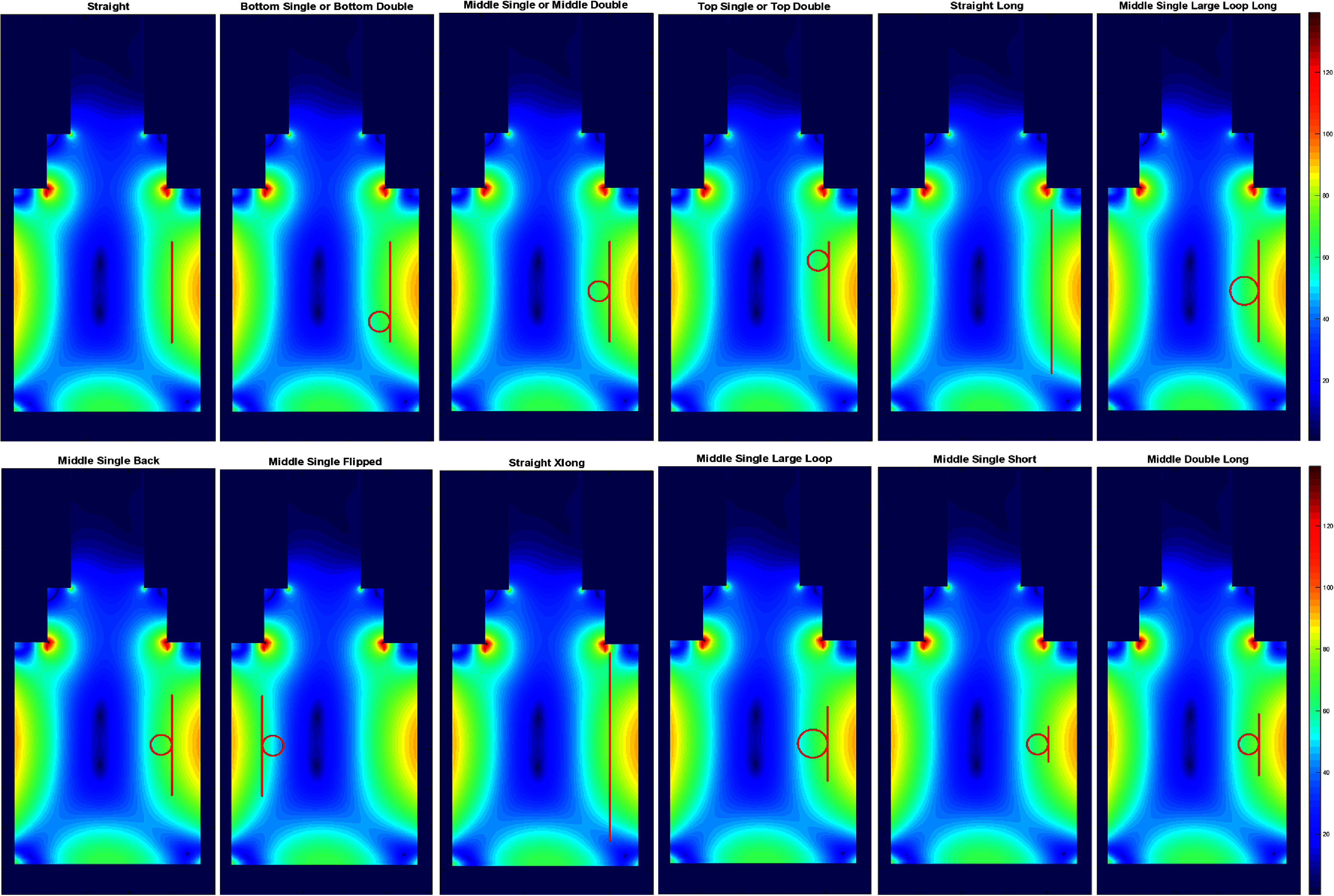
Electric field simulations (see text) for the ASTM phantom at *y* = 0 (i.e. a coronal midline slice) at 1.5 T showing all the different configurations of the vertical wires tested. Note that ‘lead tip’ end of the wire corresponds to the superior end of each vertically-oriented wire.

**Figure 3.**
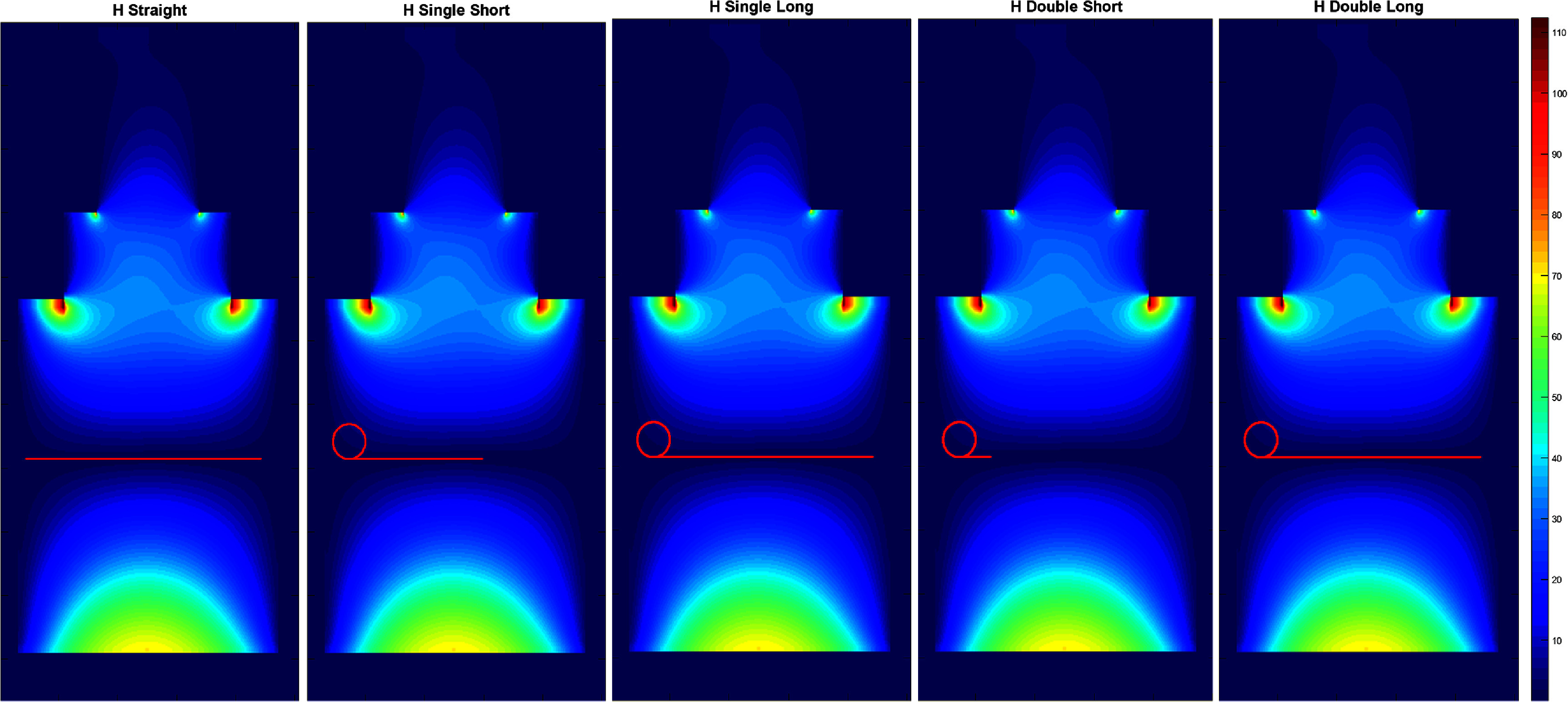
Electric field *x*-component simulations for the ASTM phantom at *y* = 0 (i.e. a coronal midline slice) at 1.5 T showing all the different configurations of the horizontal wires tested. Note that ‘lead tip’ end of the wire corresponds to patient-right end of each horizontally-oriented wire.

For temperature rise measurements, an ASTM phantom was used to house the different configurations of the insulated wire. The ‘IPG’ end of the wire was insulated/capped with a silicone gel, corresponding to the inferior end of each S/I (i.e. ‘vertically-’) oriented wires and the patient-left end of the R/L (i.e. ‘horizontally-’) oriented wires. The ‘lead tip’ end of the wire had 5 mm of the insulation removed, corresponding to the superior end of each vertically-oriented wire and patient-right end of each horizontally-oriented wire. The phantom followed the ASTM F2182-11a standard (2011) and the gel had a relative permittivity of 80 and a conductivity of 0.47 S m^−1^. Coronal views of the wire configurations are shown schematically in figures 2 and 3 for vertically- and horizontally-oriented straight segments, respectively. The vertical wire paths in figure 2 are superimposed on the magnitude of the square root of the sum of squares of the ${E_x},{\text{ }}{E_y}{\text{ }}$ and ${E_z}{\text{ }}$ components of the calculated electric field, and the horizontal paths are superimposed on the magnitude of the ${E_x}{\text{ }}$ component as shown in figure 3. Each wire configuration either included zero loops (i.e. a straight wire), one loop, or two loops. The S/I center of each wire configuration was placed at isocenter. All loops and straight sections were coplanar on a mid-line coronal plane of the scanner (*y* = 0). The loop diameters are provided in table 1, with most loops approximately 5 cm in diameter, and larger loops approximately 7 cm.

**Table 1.** Total length and total height (Δ*z*) for all wires tested. Note ‘H’ denotes horizontal wires (i.e. oriented R/L), while all the rest labels denote vertical wires (i.e. oriented S/I).

	Total length (cm)	Total height (cm)	# of Loops	Diameter of loops (cm)
Straight	25	25	0	—
Bottom single	40.7	25	1	5
Middle single	40.7	25	1	5
Top single	40.7	25	1	5
Bottom double	56.4	25	2	5
Middle double	56.4	25	2	5
Top double	56.4	25	2	5
Straight long	40.7	40.7	0	—
Middle single large loop long	47	25	1	7
Middle single back	40.7	25	1	5
Middle single flipped	40.7	25	1	5
Straight xlong	47	47	0	—
Middle single large loop	40.7	18.7	1	7
Middle single short	25	9.3	1	5
Middle double long	47	15.5	2	5
H straight	40	40	0	—
H single short	40	25.5	1	5.5
H single long	54.8	8.2	1	5.5
H double short	40	40	2	5.5
H double long	72.1	40	2	5.5

### 3.2. Temperature measurements

Using a 1.5 T scanner (HDx 16.0, GE HealthCare, Chicago, IL, USA), temperature measurements were taken at the lead tips for all 20 different wire configurations. Temperature measurements were collected during an axial T2-weighted fast spin echo pulse sequence using a Fluoroptic® thermometer (Luxtron model FOT Lab Kit, Lumasense Technologies, Santa Clara, CA, USA) with a resolution of 0.1 °C and sampling rate of 10 s^−1^. For each wire, the table position was set so the middle of the ‘torso’ was at the isocenter. Three temperature probes (model STF-2) were used. One was placed as a reference away from the lead tip and two were placed less than 1.0 mm from the lead tip.

As in Bardwell Speltz *et al* (2024), a transmit gain (TG) correction was used to adjust the temperature measurements. This correction equalizes slight variation in flip angle across the various wire experiments. As previously reported in Bardwell Speltz *et al* (2024), the effects of the TG correction were minor.

### 3.3. Simulated, spatially varying electric field

The simulated lead tip voltages were calculated using a spatially varying, simulated electric field **E**, along with equation (1) and the transfer function from either equation (6) for the simple exponential model or equation (7) for the adapted transmission line model via a discrete summation. The electric field generated by the 1.5 T body transmit coil was simulated with an ASTM phantom using Sim4Life (ZMT, Zurich, Switzerland) (Tarasek *et al* 2022, Bardwell Speltz *et al* 2024), which gave a spatial map of the complex electric field **E**. We then calculated the tangential component using equation (2). The RF isocenter was set to match the experimental location (middle of the ‘torso’). The *z*-direction contains the strongest E-field, with the larger values being along the sides of the phantom. We observed that along many paths of the simulated E-field in the phantom, $\left| {{E_z}} \right| \gg {\text{ }}\left| {{E_x}} \right|{\text{ }}$ and $\left| {{E_z}} \right| \gg {\text{ }}\left| {{E_y}} \right|$. This indicated that the ${E_z}$ term dominated much of the line integral. For reference, we also repeated the calculation with the long-wavelength approximation, $h = 1$, but note that this calculation used the simulated electric field, which is more sophisticated than the simplifying assumption of a linear electric field made in the appendix.

#### 3.4. Statistical analysis

**Figure 4.**
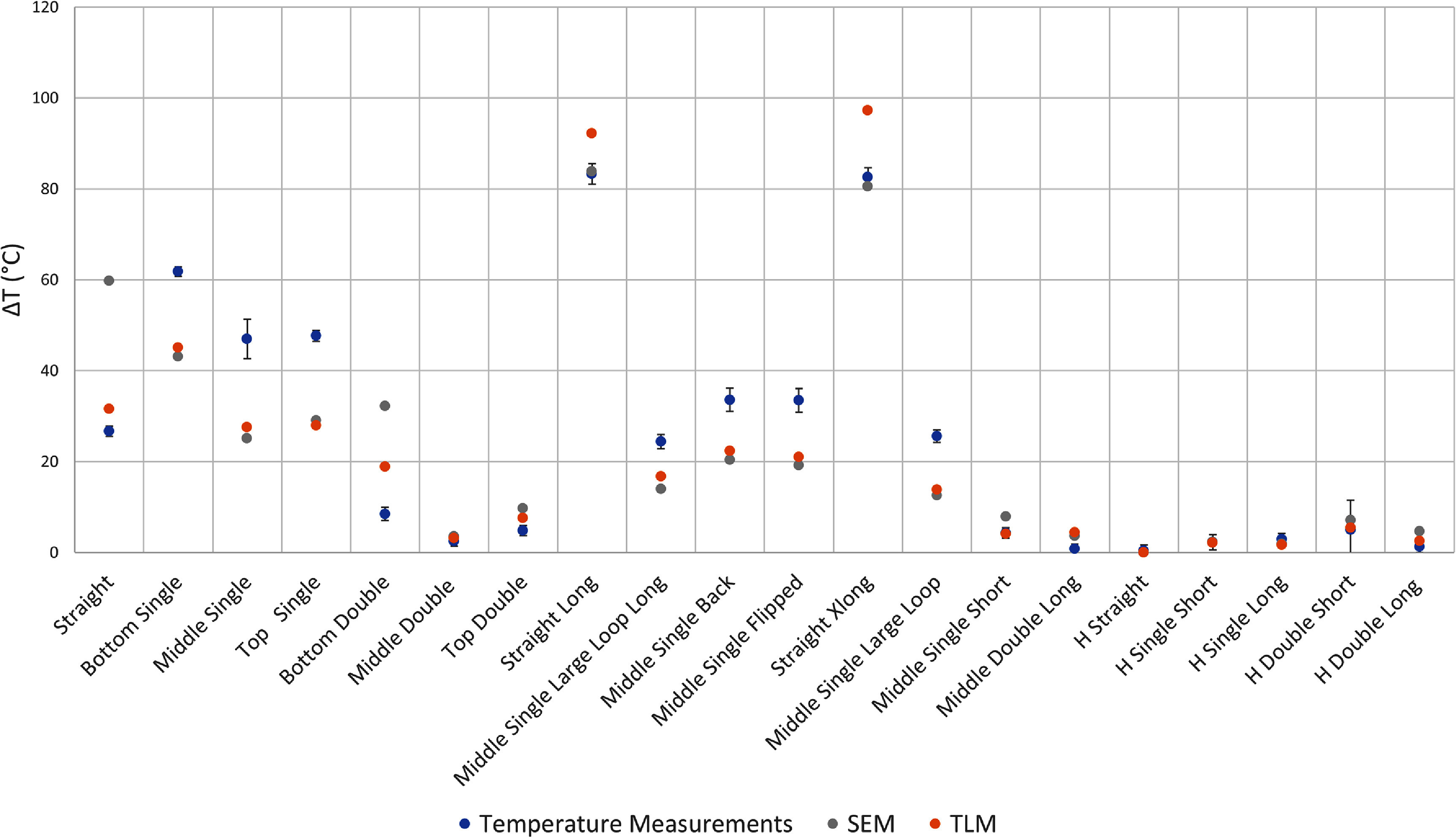
Experimental results of measured temperature rise for all the wires tested as well as the predicted temperature rise ${{\Delta }}T$ from the adapted transmission line model (TLM) using an optimized $\Gamma $ and the simple exponential model (SEM).

**Figure 5.**
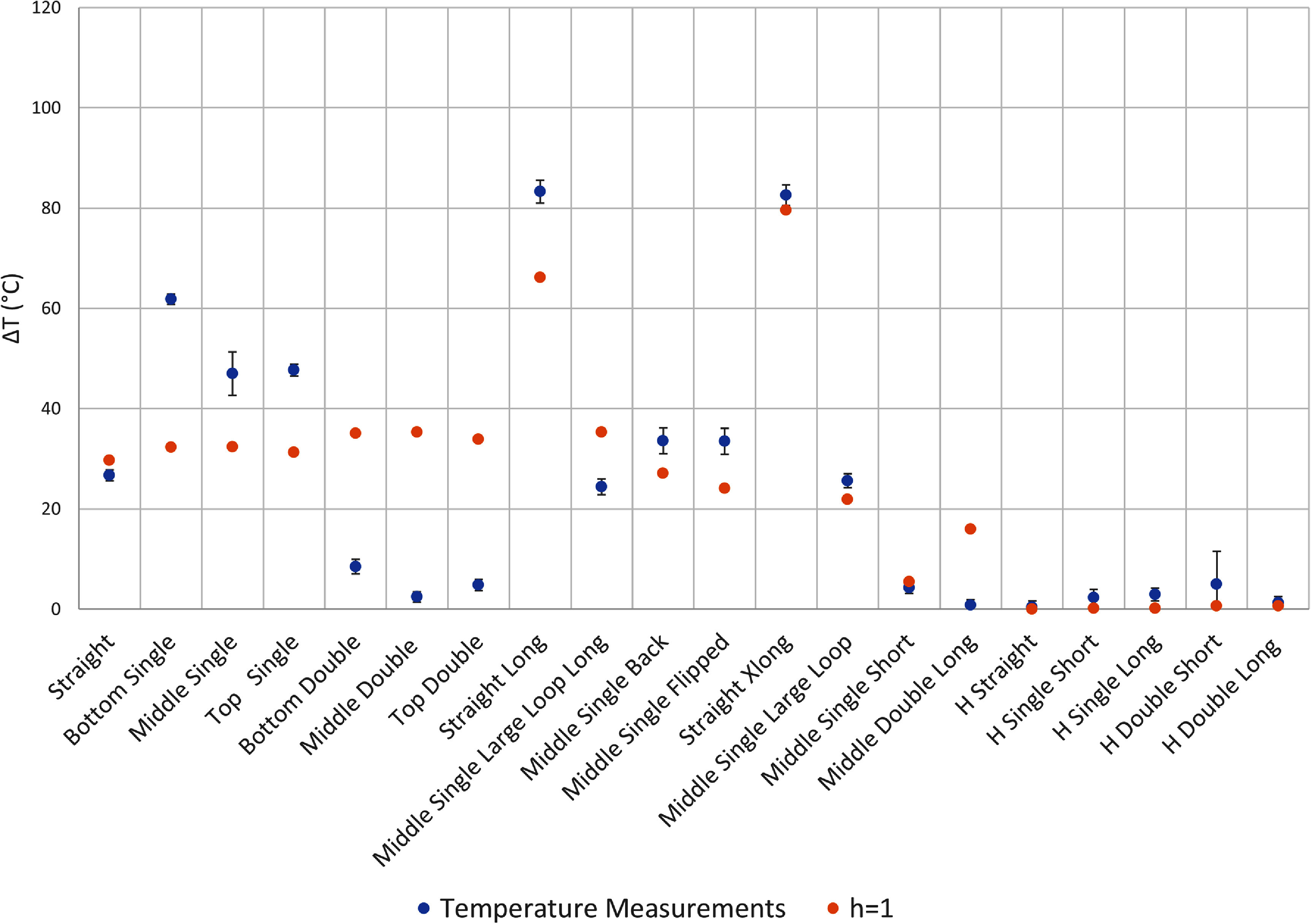
Experimental results of measured temperature rise for all the wires tested as well as the predicted temperature rise ${{\Delta }}T$ from $h\left( l \right) = 1$.

We identified three sources of error in our temperature measurements. The first source of error arises from the variation in temperature rise recorded by the two different fluoroptic probes positioned at the lead tip. The second source of error stems from any fluctuation in TG values calibrated during the scanner’s automatic prescan workflow. The third source of error is the standard deviation of the baseline temperature recorded before any RF power was applied. The overall error, represented by the error bars in figures 4, 5, 7, and 8 was calculated by taking the square root of the sum of the squares of these three types of error.

A single scaling factor was fitted to the temperature rise data for each transfer function model prediction in order to minimize root mean square error (RMSE) over the entire set of 20 wires. As described in Theory, the adapted transmission line model includes an additional parameter for the reflection coefficient. Since absolute truth data was not available, we used the Akaike Information Criterion (AIC) to compare the models, accounting for the different number of free parameters. The AIC determines how well a model fits data with a penalty term that is determined by the number of free parameters used. As the number of free parameters increases, so does the penalty term. The AIC was calculated using ordinary least squares, as outlined in (Banks and Joyner 2017). Meanwhile, Bland–Altman plots were generated to compare the SEM, TLM, and $h = 1$ estimation with the measured temperature to help visualize the difference between the predicted and measured temperature rise.

## 4. Results

As determined from equation (4), the wavenumber of the medium (i.e. ASTM gel) is (14.4974–8.1746*i*) rad m^−1^. The wavenumber of the insulator is 2.0301 rad m^−1^ calculated from equation (4), assuming $\sigma = 0.$ The King wavenumber is (6.7454–0.6840*i*) rad m^−1^ calculated from equation (5).

**Figure 6.**
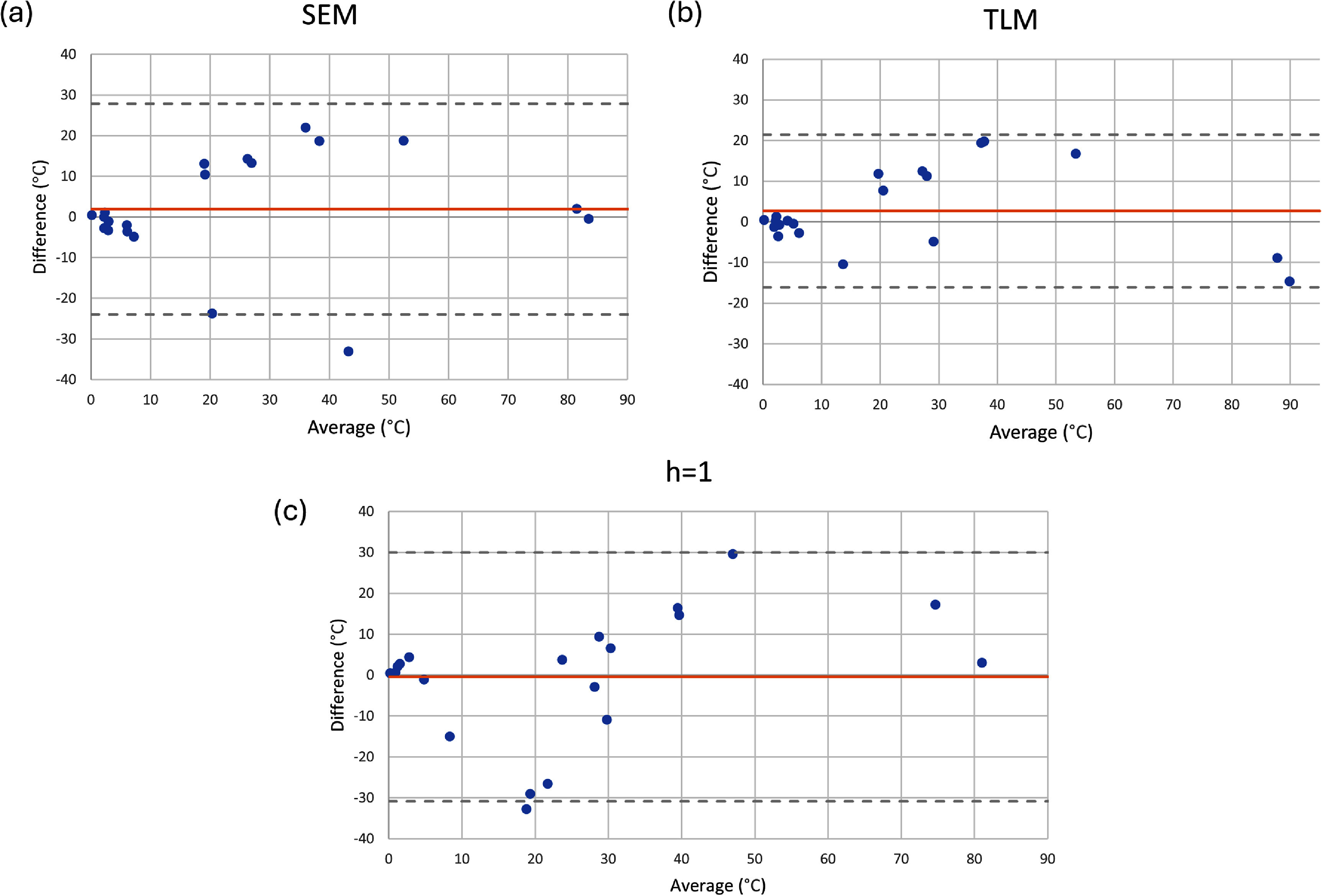
Bland–Altman plots showing the difference versus the average between the model and the measured temperature for the (a) SEM, (b) TLM, and (c) $h = 1$. The orange line gives the mean of the differences and the gray dashed lines give the mean of the differences plus or minus 1.96 times the standard deviation of the differences.

Figure 4 compares the predictions of the simple exponential model and the adapted transmission line model against the measurements for the 20 different wires. The fitted reflection coefficient Γ that appears in equation (7) was determined to be 0.2403. Figure 5 shows a plot of the temperature rise versus the experimental data for the $h = 1$ approximation. Figure 6 shows the Bland–Altman plots for the three transfer function models, compared to the measured temperature data.

**Figure 7.**
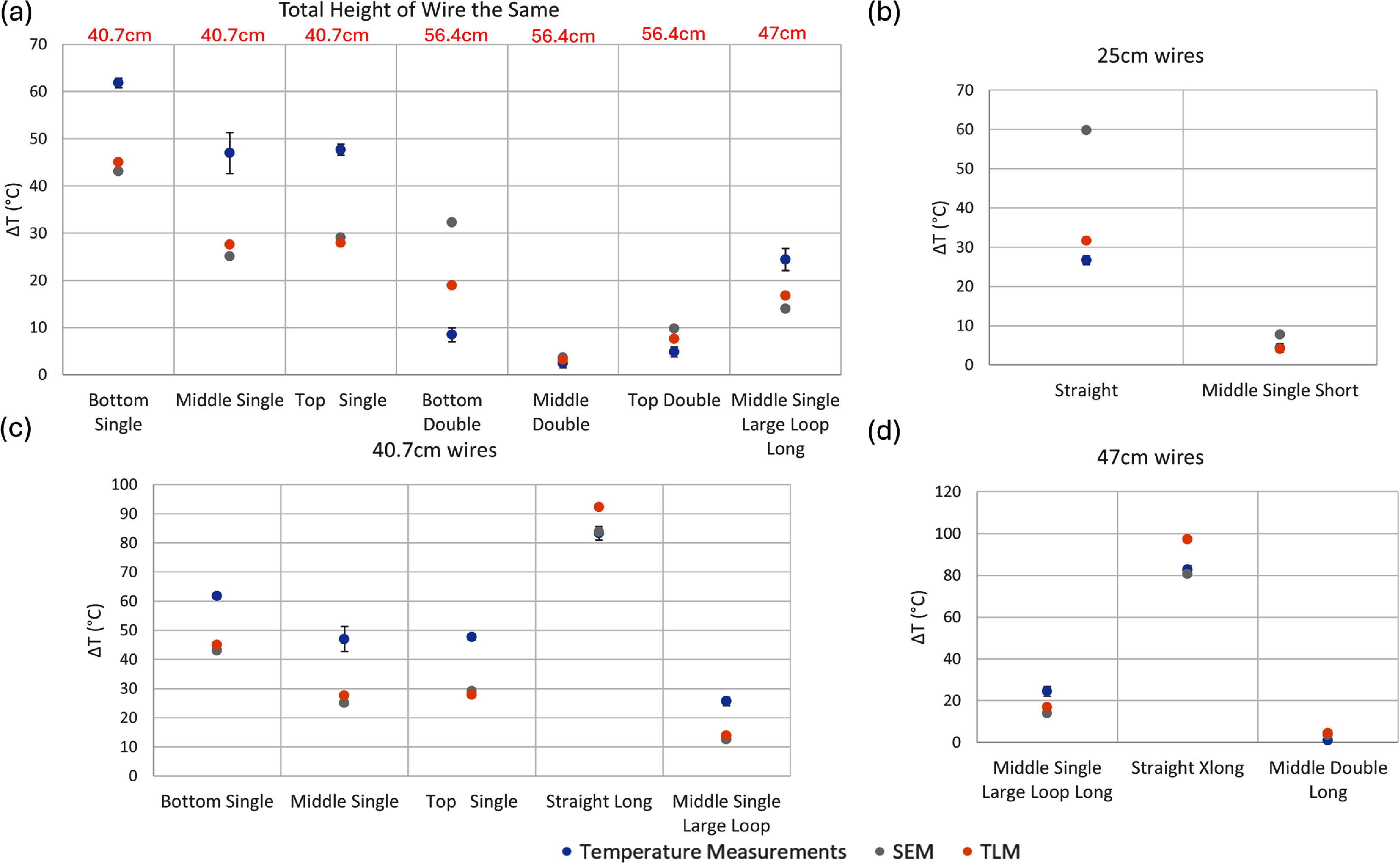
Experimental results for all vertical wires tested as well as the predicted temperature rise ${{\Delta }}T$ from both the adapted transmission line model (TLM) using an optimized $\Gamma $ and the simple exponential model (SEM) for various groups including (a) vertical wires with the same total height with the length of each wire shown in red text, (b) vertical wires that were 25 cm in length (c) vertical wires that were 40.7 cm in length (d) vertical wires that were 47 cm in length.

To better understand the results, we plot some of the data in figure 4 separately in individual subplots, especially because the maximum temperature rise has a large dynamic range across the experiments. To assess the influence of overall wire length on heating, we conducted group comparisons that controlled for either the total height (i.e. S/I extent of the wire configuration, regardless of any loop(s)) of the wire Δ*z*, or the total wire length *d*. The results of these group comparisons are presented in figure 7, which includes: (a) vertically-oriented wires with the same net height of 25 cm, (b) vertically-oriented wires with total length of 25 cm, (c) vertically-oriented wires with total length of 40.7 cm, and (d) vertically-oriented wires with total length of 47 cm.

Table 2 provides a comparison of the RMSE and the AIC for the simple exponential model, the adapted transmission line model and the $h = 1$ approximation. Among these, the adapted transmission line model with the optimized Γ provided a better fit, achieving the lowest RMSE and AIC values, indicating better predictive accuracy even with the additional free parameter, consistent with the result of Bardwell Speltz *et al* (2024). As expected, the simple exponential model outperformed the $h = 1$ approximation, without adding any additional free parameters.

**Table 2.** Statistical results for $h\left( l \right) = 1$, the simple exponential model (SEM), and adapted transmission line model (TLM) fits. RMSE = root mean square error, AIC = Akaike Information Criterion.

	*h* = 1	SEM	TLM
Number of free parameters	1	1	2
RMSE	15.52	13.35	9.95
AIC	28.89	25.87	22.00

**Figure 8.**
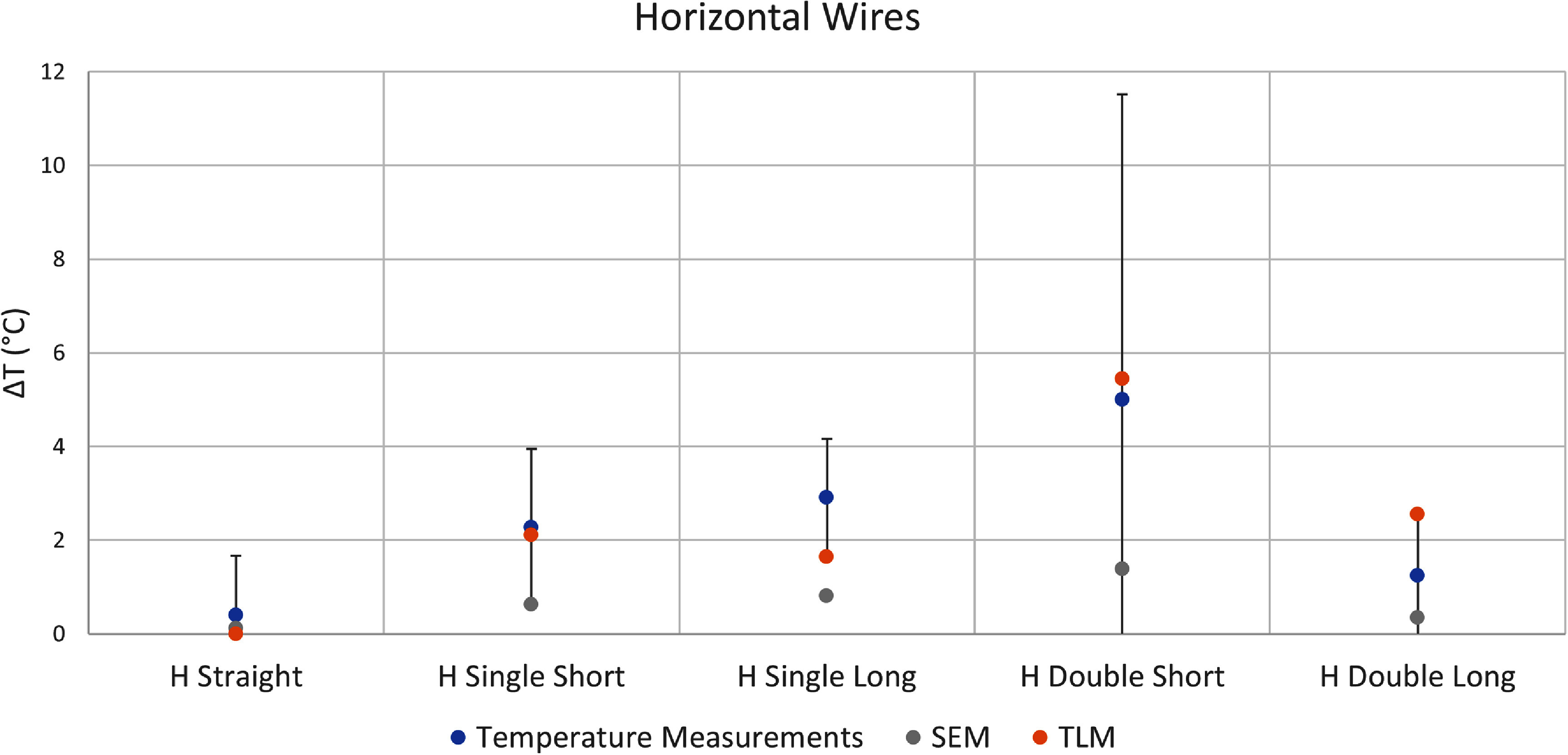
Experimental results for all horizontal wires tested as well as the predicted temperature rise ${{\Delta }}T$ from both the adapted transmission line model (TLM) using an optimized $\Gamma $ and the simple exponential model (SEM). Note the range of the temperature rise is quite different from figure 4.

In figure 8, the temperature rise results of the simple exponential model, the adapted transmission line model, and the corresponding experimental data are shown for all the horizontally-oriented wires. Note that the error bars appear relatively large because the measured temperature rise was small (5 °C or less) compared to the vertically-oriented wires. Hence, the vertical axis of the plots in figure 7 has a much larger range compared to figure 8. Comparing the three cases with the same wire length (horizontally-oriented (H) straight, H single short, and H double short whose configurations are shown in figure 3), it is apparent that adding loops increased the temperature for the horizontal wires.

## 5. Discussion

We considered three transfer function models for lead tip heating. The simplest model uses a constant transfer function *h* = 1, so it does not account for wavelength or lossy effects because it lacks dependence on the complex wavenumber *k*. In this sense, it can be considered a long-wavelength approximation. This model was included here primarily because, in conjunction with a simplifying assumption of linearly-varying electric field, it allows for the analytical derivation provided in the appendix, which may aid physical intuition.

Next, the SEM described by equation (6) captures wavelength and loss effects via the King wavelength, but it does not account for reflection at the end of the lead. Not surprisingly, it outperformed the *h* = 1 model (see table 2), although it has no additional free parameters. The adapted TLM of equation (7) accounts for reflection by adding one additional free parameter, the reflection coefficient ${{\Gamma }}$. Notably, the value of ${{\Gamma }}$ fitted from the data measure here closely matched the value reported in (Bardwell Speltz *et al* 2024), which used the same type of wire.

As illustrated by figure 4 and table 2, both the simple exponential model and the adapted transmission line model appear to effectively account for the different loop configurations well, with the adapted transmission line model providing a better quantitative fit according to the AIC statistical metric, which accounts for the number of free parameters.

Notably, neither the SEM or the TLM require modification or additional terms to equation (1) to account for the presence of loops. This can best be understood considering equation (8). Because the simulated electric field has a non-zero curl, the magnetic field is implicitly accounted for and is integrated within the model, obviating the need for the magnetic field to be considered separately. The result shows the robustness of these simple analytical models in handling more complex wire geometries beyond straight wires, especially those involving loops.

A trend that is apparent in much of figure 4 and throughout figure 7 is that the introduction of loops and increasing their number of turns *N* generally *reduce* heating (with some exceptions discussed in the next paragraph) consistent with the findings of previous studies (Baker *et al* 2005, Mattei *et al* 2006, Golestanirad *et al* 2017, Bhusal *et al* 2021, Vu *et al* 2023). This trend can be understood based on the simplified calculation provided in appendix, including the scaling relation with the number of loop turns *N*, and aligns well with the concept of electrical length introduced in section 2.2. That is, the portion of the wire used for the loops contributes a relatively small voltage of its own compared to a straight section of the same length, while attenuating the transfer function (see figure 1) farther along the path of the line integral in equation (1). This attenuation becomes more pronounced the larger the negative imaginary part of the complex wavenumber.

This reduction in heating with loop(s) is not universal as reported in Akter *et al* (2024), so caution should be exercised in applying this rule in practice. Note most of the wires shown in figure 4 and all wires in figure 7 were positioned vertically. However, guided by the underlying theory, we designed an experiment in which heating was expected to increase with the introduction of loops. The wires shown in figure 3 were positioned horizontally, where ${E_x} \approx 0$. As a result, the line integral of the straight segments contribute negligible voltage, so heating is expected to *increase* with the introduction of loops. This theoretical prediction and experimental results are illustrated in figure 8. Furthermore, as additional loops of the same size were added, the heating effect continues to intensify. The contrasting behavior between vertical and horizontal wires underscores the need for careful consideration of wire geometry when designing and evaluating lead heating for implanted medical devices.

For the vertically-oriented wires, in addition to the number of the loops, the location of the loop also effects overall heating. Wires with loops positioned further from the lead tip (bottom loops) heat more than wires with equivalent loops near the lead tip (top loops). Again, this can be explained with the electrical length concept, because the top loops add path length and attenuate the transfer function over the entire remaining (upstream) straight section of the wire, while the bottom loops do not affect the electrical distance to the lead tip for the preceding (downstream) straight portion of the wire. Given the generally decaying nature of $\left| {h\left( l \right)} \right|$ apparent on figure 1, lead-tip heating is expected to decrease when a loop is located closer to the lead tip (on the superior side of the phantom in these experiments).

Considering the magnitude $\left| {h\left( l \right)} \right|$ alone does not explain the result that loops in the middle of the wire achieved the lowest temperature. This was observed among the first three cases (bottom, middle, top single; 40.7 cm wire length) and the second set of three cases (bottom, middle, top double; 56.4 cm wire length) in figure 7(a), both in experiment and simulation. One possible explanation can come from the phase of the transfer function $\measuredangle h\left( l \right)$, also plotted in figure 1(b). In the mentioned cases, the wire lengths were comparable (within 21%) to $\lambda /2 = 46.6\;{\text{cm}}$. This implies that the contributions to the complex path integral equation (1) from the top and the bottom regions of the wire would have roughly opposed phases and tend to cancel partially. Inserting a loop in the middle of the wire suppresses the contribution of that section of the wire as explained above, leaving the integral dominated by the two end regions, leading to reduction in the summed voltage. While more experiments are needed for further verification, this consideration may offer a way to suppress resonant heating when the wire length approaches half wavelength.

There are several limitations to this study. The measurements were limited to straight and circular loop segments in insulated wires that were not actual device leads, with one end capped with an insulator. The physical and electrical properties of the wires can differ from those of clinical leads. While this setup serves as a reasonable approximation for capped, abandoned leads, it does not fully represent various clinical scenarios, such as leads connected to IPGs, leads capped with materials other than insulators (Wang *et al* 2022), or helical leads (Liorni *et al* 2018). Additionally, only a limited number of configurations of loops (e.g. all circular) were investigated here. Due to geometrical limitation of the phantom, we only studied loops aligned with the coronal plane of the scanner bore. More complex lead geometries, including those with bends or irregular shapes, were not considered and might exhibit different heating characteristics. Lastly, the study’s findings were obtained only in the 1.5T MRI environment. Different field strengths could result in different heating patterns, especially when resonant lengths match the wire length.

The limitation of our experimental setup such as the use of simplified wire and limited phantom configuration, suggest that further research is necessary. Our future work could include replicating these experiments at 3 T and 7 T, systematically studying the effect loops have on resonant length, exploring more complex wire geometries, and testing *in vivo* scenarios to validate these findings and refine our predictive models. Because $h = 1$ is a long-wavelength approximation, we expect that its validity will decrease at fields above 1.5 T compared to the simple exponential model and the adapted transmission line model, consistent with results reported in Bardwell Speltz *et al* (2024).

## 6. Conclusions

This study investigated the maximum temperature rise at the lead tips of various insulated wires placed in a phantom setup at 1.5 T, considering different orientation, loop placements and numbers of loops. We compared our experimental results with predictions from the simple exponential model, the adapted transmission line model, and the long wavelength approximation of $h = 1$. Overall, all models qualitatively captured trends in lead tip temperature rise. Quantitatively, the adapted transmission line model provided the most accurate predictions, as evidenced by the lowest AIC values as well as the tightest spread in a Bland-Altman plot, followed by the simple exponential model.

In conclusion, this study provides insight into how wire looping affects lead tip RF heating and demonstrates potential application of the transfer function-based theoretical models for managing the heating risks.

## Figures and Tables

**Figure A1.**
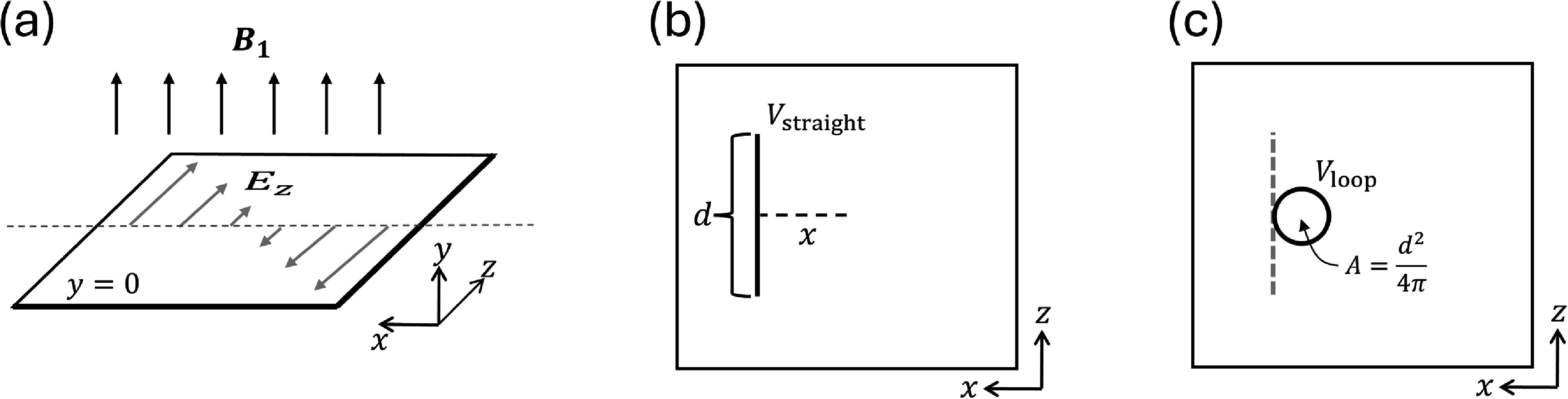
(a) Electric field on a coronal plane induced by a uniform RF B1 field. (b) Straight wire on the same plane displaced by *x* from the isocenter. *V*_straight_ denotes the induced voltage in the wire. (c) Circular loop formed by the same length of wire where the induced voltage is *V*_loop_.

## Data Availability

All data that support the findings of this study are included within the article (and any supplementary information files).

## Appendix

Here we examine the relative contributions to lead tip voltage from a straight wire and loops of the same total wire length, under simplified conditions. The derived equations were not used to calculate the voltage shown in Results. Consider a uniform B1 field whose magnitude in the *y*-direction is $b{1_{\text{0}}}$ (figure A1(a)). Because equation (8) is evaluated in the laboratory (non-rotating) frame, $\left| {{\text{d}}{B_y}/{\text{d}}t} \right| = \omega { }b{1_{\text{0}}}$, where $\omega /2\pi $ is the Larmor frequency. For simplicity, let us consider a mid-line coronal plane (i.e. *y*= 0). Then, also from equation (8), projected along the *y* axis, the *z*-component of electric field can be written as:

\begin{align*}{E_z} = {\text{ }}x{\text{ }}\omega {\text{ }}b{1_{\text{0}}} + {\text{c }}\end{align*}

in a region where *E_z_* dominates *E_x_* (see, for example, figure 4 of Bardwell Speltz *et al* 2024). Here we have omitted the time-dependent sinusoidal factor along with a uniform phase because they do not affect the magnitude of the line integration in equation (1). We will set the constant *c* in equation (A1) equal to zero, which is consistent with the well-known result of a straight wire that is placed midline produces negligible heating. This choice is also consistent with standard EM simulations (e.g. figure 4 in Bardwell Speltz *et al* 2024) showing *z*-component of electric field is zero in a midline coronal plane when *x* = 0.

The transfer functions given by equation (6) reduce to $h\left( l \right) \approx 1{\text{ }}$ when ${\text{ }}\left| k \right|d \ll 1$, which occurs when the wire length *d* is much shorter than the King wavelength $\lambda = 2\pi /{\text{Re}}\left( k \right)$. (The same is true for equation (7), provided that $\Gamma \ne 1$). If we do make the simplifying assumption that $h\left( l \right) = 1$, then for a vertically-oriented (i.e. in the *z*-direction), straight wire of length *d* that is displaced right-left from midline by a distance *x* (figure A1(b)), equation (1) reduces to.

\begin{align*}{V_{{\text{straight}}}} = x{ }d{ }\omega { }b{1_{\text{0}}}.\end{align*}

If this straight wire is bent into a circular loop (figure A1(c)), then the area *A* of the resulting loop is

\begin{align*}A = { }\frac{{{d^2}}}{{4\pi }}.\end{align*}

According to Faraday’s law of induction, the voltage induced in the loop is ${V_{{\text{loop}}}} = A{\text{ }}\omega {\text{ }}b{1_{\text{0}}}$, and combining equations (A2) and (A3) yields

\begin{align*}\left| {\frac{{{V_{{\text{loop}}}}}}{{{V_{{\text{straight}}}}}}} \right| = \frac{{{ }d}}{{4\pi \left| x \right|}}.\end{align*}

Note that the ratio in equation (A4) is independent of both main magnetic and RF field strength (i.e. respectively), and instead only depends on geometrical factors. (Recall, however, that at higher field strengths the long-wavelength approximation $h = 1$ breaks down). Also note that the straight wire produces greater voltage than the loop whenever $\left| x \right| &gt; \frac{{{\text{ }}d}}{{4\pi {\text{ }}}}$, which is usually satisfied unless the straight wire is intentionally placed along the midline.

Equations (A2)–(A4) can be readily generalized to the case of the wire bent into a circular loop with *N* turns, again holding its total length *d* constant. Then the area *A* of the resulting loop becomes

\begin{align*}A = {\text{ }}\frac{{{d^2}}}{{4\pi {N^2}}}\end{align*}

and the voltage induced in the loop is ${V_{{\text{loop}}}} = N{\text{ }}A{\text{ }}\omega {\text{ }}b{1_{\text{0}}}$, yielding

\begin{align*}\left| {\frac{{{V_{{\text{loop}}}}}}{{{V_{{\text{straight}}}}}}} \right| = \frac{{{ }d}}{{4\pi N{ }\left| x \right|}}.\end{align*}

From equation (A6), we can see the inverse linear dependence with the number of turns *N* in this simplified model. Also note that the condition $\left| x \right| &gt; \frac{{{\text{ }}d}}{{4\pi N{\text{ }}}}$ becomes even easier to satisfy as *N* increases.

## References

[pmbadcc73bib1] Akter M K, Guo R, Islam M Z, Zheng J, Kainz W, Long S A, Chen J (2024). Effect of strain relief loop position on the RF-induced heating of active implantable medical devices at 1.5-T MRI. IEEE Trans. Electromagn. Compat..

[pmbadcc73bib2] Arfken G B (1985). Mathematical Methods for Physicists.

[pmbadcc73bib3] ASTM F2182-11a (2011). Standard Test Method for Measurement of Radio Frequency Induced Heating on or near Passive Implants during Magnetic Resonance Imaging.

[pmbadcc73bib4] Baker K B, Tkach J, Hall J D, Nyenhuis J A, Shellock F G, Rezai A R (2005). Reduction of magnetic resonance imaging-related heating in deep brain stimulation leads using a lead management device. J. Neurosurg..

[pmbadcc73bib5] Banks H T, Joyner M L (2017). AIC under the framework of least squares estimation. Appl. Math. Lett..

[pmbadcc73bib6] Bardwell Speltz L J, Lee S K, Shu Y, Tarasek M R, Trzasko J D, Foo T K F, Bernstein M A (2024). Modeling and measurement of lead tip heating and resonant length for implanted, insulated wires. Magn. Reson. Med..

[pmbadcc73bib7] Bhusal B, Keil B, Rosenow J, Kazemivalipour E, Golestanirad L (2021). Patient’s body composition can significantly affect RF power deposition in the tissue around DBS implants: ramifications for lead management strategies and MRI field-shaping techniques. Phys. Med. Biol..

[pmbadcc73bib8] Feng S, Qiang R, Kainz W, Chen J (2015). A technique to evaluate MRI-induced electric fields at the ends of practical implanted lead. IEEE Trans. Microw. Theory Tech..

[pmbadcc73bib9] Golestanirad L (2019). RF-induced heating in tissue near bilateral DBS implants during MRI at 1.5T and 3T: the role of surgical lead management. Neuroimage.

[pmbadcc73bib10] Golestanirad L, Angelone L M, Iacono M I, Katnani H, Wald L L, Bonmassar G (2017). Local SAR near deep brain stimulation (DBS) electrodes at 64 and 127 MHz: a simulation study of the effect of extracranial loops. Magn. Reson. Med..

[pmbadcc73bib11] Jackson J D (1999). Classical Electrodynamics.

[pmbadcc73bib12] Jiang F, Bhusal B, Nguyen B, Monge M, Webster G, Kim D, Bonmassar G, Popsecu A R, Golestanirad L (2023). Modifying the trajectory of epicardial leads can substantially reduce MRI-induced RF heating in pediatric patients with a cardiac implantable electronic device at 1.5T. Magn. Reson. Med..

[pmbadcc73bib13] King R W, Lee K M, Mishra S R, Smith G S (1974). Insulated linear antenna: theory and experiment. J. Appl. Phys..

[pmbadcc73bib14] Liorni I, Neufeld E, Kühn S, Murbach M, Zastrow E, Kainz W, Kuster N (2018). Novel mechanistic model and computational approximation for electromagnetic safety evaluations of electrically short implants. Phys. Med. Biol..

[pmbadcc73bib15] Liu J, Zheng J, Wang Q, Kainz W, Chen J (2018). A transmission line model for the evaluation of MRI RF-induced fields on active implantable medical devices. IEEE Trans. Microw. Theory Tech..

[pmbadcc73bib16] Mattei E (2006). MRI induced heating of pacemaker leads: effect of temperature probe positioning and pacemaker placement on lead tip heating and local SAR. Conf. Proc. IEEE Eng. Med. Biol. Soc..

[pmbadcc73bib17] Milton A, Stegun I A (1972). Handbook of Mathematical Functions.

[pmbadcc73bib18] Nitz W R, Oppelt A, Renz W, Manke C, Lenhart M, Link J (2001). On the heating of linear conductive structures as guide wires and catheters in interventional MRI. J. Magn. Reson. Imaging.

[pmbadcc73bib19] Nyenhuis J A, Park S M, Kamondetdacha R, Amjad A, Shellock F G, Rezai A R (2005). MRI and implanted medical devices: basic interactions with an emphasis on heating. IEEE Trans. Device Mater. Reliab..

[pmbadcc73bib20] Park S M, Kamondetdacha R, Nyenhuis J A (2007). Calculation of MRI-induced heating of an implanted medical lead wire with an electric field transfer function. J. Magn. Reson. Imaging.

[pmbadcc73bib21] Tarasek M R, Shu Y, Kang D, Tao S, Gray E, Huston J, Hua Y, Yeo D T B, Bernstein M A, Foo T K (2022). Average SAR prediction, validation, and evaluation for a compact MR scanner head-sized RF coil. J. Magn. Reson. Imaging.

[pmbadcc73bib22] Vu J, Bhusal B, Rosenow J M, Pilitsis J, Golestanirad L (2023). Effect of surgical modification of deep brain stimulation lead trajectories on radiofrequency heating during MRI at 3T: from phantom experiments to clinical implementation. J. Neurosurg..

[pmbadcc73bib23] Wang Y (2022). Magnetic resonance conditionality of abandoned leads from active implantable medical devices at 1.5 T. Magn. Reson. Med..

[pmbadcc73bib24] Yeung C J, Susil R C, Atalar E (2002a). RF safety of wires in interventional MRI: using a safety index. Magn. Reson. Med..

[pmbadcc73bib25] Yeung C J, Susil R C, Atalar E (2002b). RF heating due to conductive wires during MRI depends on the phase distribution of the transmit field. Magn. Reson. Med..

